# Analysis of the association between osteoporosis and muscle strength in Korean adults: a national cross-sectional study

**DOI:** 10.1186/s41043-023-00443-w

**Published:** 2023-09-12

**Authors:** Ji-Young Choi, Young-Mo Yang

**Affiliations:** 1 Department of Food and Nutrition, College of Natural Science and Public Health and Safety, Chosun University, Gwangju, Republic of Korea; 2 Department of Pharmacy, College of Pharmacy, Chosun University, 309 Pilmun-daero, Dong-gu, Gwangju, 61452 Republic of Korea

**Keywords:** Osteoporosis, Hand grip strength, Aging, Body mass index, Renal function, Koreans, Cross-sectional study

## Abstract

**Background:**

This study aimed to examine the associations between osteoporosis and hand grip strength (HGS), a surrogate marker of muscular strength, among Korean adults stratified by body mass index (BMI), age, and renal function.

**Methods:**

This study was conducted using the data obtained from the Korea National Health and Nutrition Examination Survey 2015–2019, a cross-sectional and nationally representative survey performed by the Korea Centers for Diseases Control and Prevention.

**Results:**

Of the 26,855 subjects included in this study, those with low muscle strength (LMS) and normal muscle strength were showed in 4,135 (15.4%) and 22,720 (84.6%) subjects, respectively. The osteoporotic subjects had a higher prevalence rate for LMS than those without osteoporosis after adjusting for age [odds ratio (OR), 1.684; 95% confidence interval (CI), 1.500–1.890). The subjects with osteoporosis and BMI < 18.5 kg/m^2^ also had a higher prevalence rate for LMS after adjusting for age compared to those with non-osteoporosis and BMI < 18.5 kg/m^2^ (OR, 1.872; 95% CI, 1.043–3.359). Compared to the non-osteoporotic subjects with estimated glomerular filtration rate (eGFR) ≥ 60 mL/min/1.73 m^2^, those with osteoporosis and eGFR ≥ 60 mL/min/1.73 m^2^ had a higher prevalence rate for LMS after controlling for age and sex (OR, 1.630; 95% CI, 1.427–1.862).

**Conclusions:**

The results showed that osteoporosis was likely to contribute to an increased prevalence rate of LMS in terms of HGS. Aging, BMI, and renal function also had significant effects on the association between osteoporosis and LMS. This association is likely to assist in developing better strategies to estimate bone health in clinical or public health practice.

## Introduction

Hand grip strength (HGS), a simple measure of upper limb function, is a straightforward and useful tool for evaluating the overall status of muscle strength [1, 2]. According to previous studies, low HGS is not only associated with all-cause mortality, nutritional status, and cardiovascular, metabolic, and respiratory diseases but is also related to cognitive dysfunction and depressive symptoms [2–10]. Low HGS is also linked to low bone mineral density (BMD) and increased prevalence of fragility fractures in post-menopausal women [11–13]. In this specific group, the increased prevalence for fractures may be related to age-associated musculoskeletal diseases such as osteopenia and osteoporosis [14–16]. Muscle strength improvement through muscle contraction was reported to have a positive effect on bone health as myokines secreted from muscles affected bone acquisition, maintenance, and improvement [17].

Changes in BMD as a surrogate marker of bone health may be related to various factors such as body mass index (BMI), advanced age, decreased kidney function, glucocorticoid use, and levels of high-density lipoprotein cholesterol (HDL-C) [18–24]. Weight loss was reported to be positively associated with a decrease in BMD, especially in peri- and post-menopausal women and older men [18, 19]. BMD reduction related to aging was also shown for both men and women [20, 21]. In women, BMD dramatically decreases after menopause; however, BMD gradually decreases with age in men [21]. Additionally, chronic kidney disease (CKD), defined as kidney damage or glomerular filtration rate (GFR) < 60 mL/min/1.73 m^2^ for 3 months or more, can lead to bone disease and fractures [22, 23]. Individuals with CKD stages 3a-5 were characterized by low BMD and an approximately twofold higher prevalence of fractures compared to healthy individuals [23].

Consequently, low BMI, aging, and decreased kidney function as contributing factors for BMD reduction may play a significant role in determining the prevalence of low muscle strength (LMS) in osteoporotic patients. However, to the best of our knowledge, only a few studies have investigated the association between osteoporosis and muscle strength in Korean adults to date [13]. Therefore, this study aims to examine the association between osteoporosis and HGS, a surrogate marker of muscular strength, among Korean adults stratified by BMI, age, and renal function.

## Methods

### Study population

**Fig. 1:**
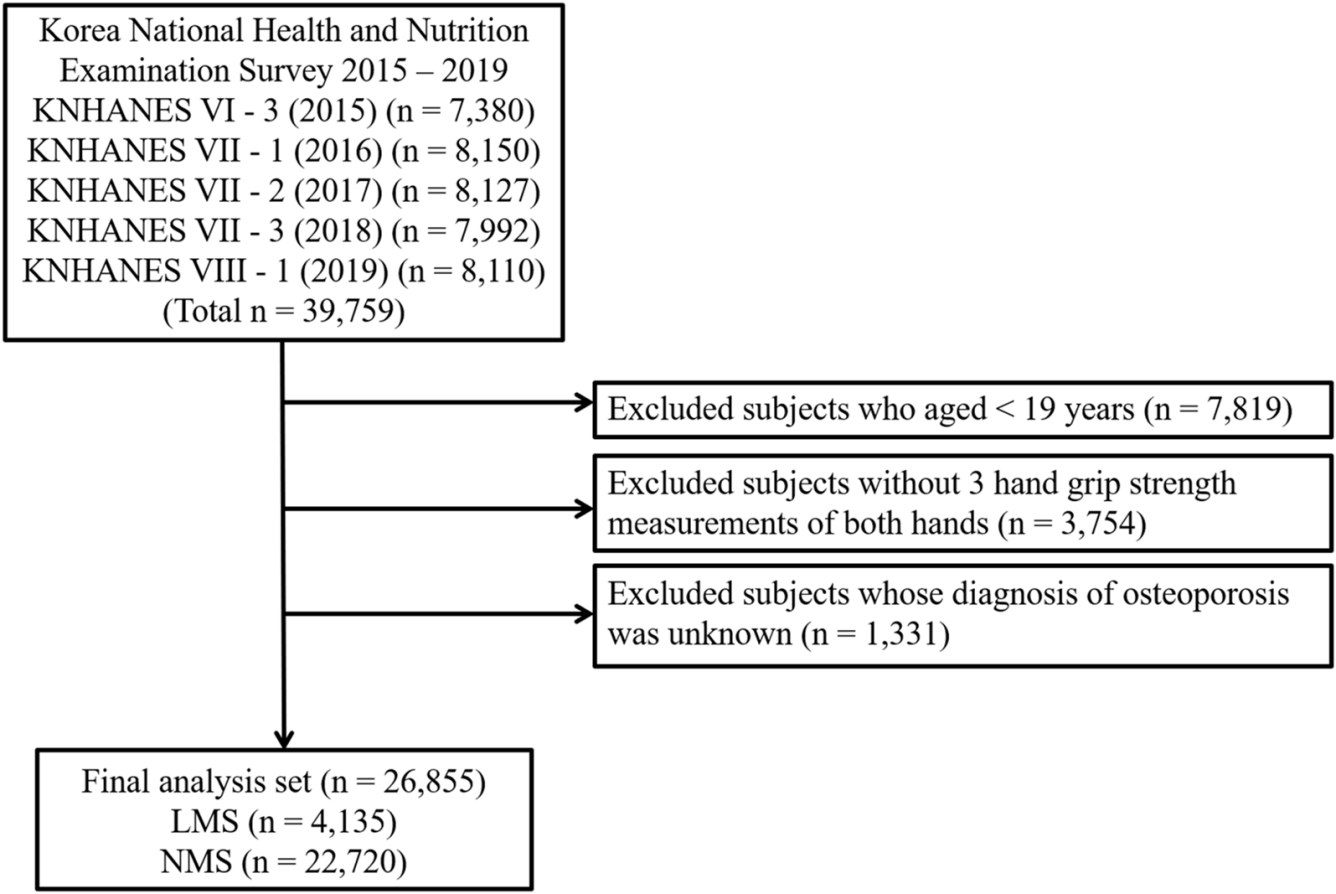
Flow chart of selecting study subjects from the Korea National Health and Nutrition Examination Survey 2015 – 2019

This study was based on the data obtained from the Korea National Health and Nutrition Examination Survey (KNHANES) 2015–2019, a cross-sectional and nationally representative survey performed by the Korea Centers for Disease Control and Prevention (KCDC). The KNHANES data included a health interview, health examination results, and answers to a nutrition survey. The data were acquired through household interviews, and standardized physical examinations were performed at mobile examination centers. The procedures for conducting the KNHANES were approved by the KCDC Institutional Review Board, and informed written consent was obtained from all survey participants. All subjects aged < 19 years were excluded. Second, those without three grip strength measurements in both hands were also excluded. Third, patients whose diagnosis of osteoporosis was unknown were not included in the analysis. Ultimately, from a total of 39,759 subjects, 26,855 adults were included in the present study (Fig. 1).

### HGS measurements

HGS was measured using a digital grip strength dynamometer (TKK 5401 Grip-D; Takei, Japan) which has an adjustable grip span. The device allows for the measurement of HGS between 5.0 and 100.0 kg, and the minimum measurement unit is 0.1 kg. During the measurement, participants stood upright with their heads up, and their arms rested in a neutral and comfortable position with elbows fully extended. The dynamometer was held in the testing hand with a 90° flexion at the index finger. Three independent measurements were performed for each hand, with the dominant hand being assessed first. The grip was squeezed for at least 3 s. While squeezing the grip, the subjects did not swing the dynamometer and were holding their breath. They rested for approximately 60 s between the measurements. The average of three measurements performed on the dominant hand was used in the statistical analysis. Based on a previous study conducted with the KNHANES data gathered in 2014 and 2015, participants were divided into two following groups: LMS and normal muscle strength (NMS). In men, LMS was defined as HGS < 28.9 kg, and NMS was defined as HGS ≥ 28.9 kg. In women, LMS was considered as HGS < 16.8 kg, and NMS was considered as HGS ≥ 16.8 kg [25].

### Anthropometric and laboratory data

Body weight and height were measured to the nearest 0.1 kg and 0.1 cm, respectively, while participants wore light indoor clothing and no shoes. BMI was calculated by dividing weight in kilograms by height in meters squared (kg/m^2^). Individuals with a BMI < 18.5 kg/m^2^ were defined as underweight, with a BMI between 18.5 and 25.0 kg/m^2^ were defined as having a normal weight, and with a BMI ≥ 25.0 kg/m^2^ were defined as overweight or obese [26].

Blood samples were collected after at least an 8-h fast, and random spot urine samples were obtained as well. The samples were processed accordingly, immediately refrigerated, and transported in a cold storage environment to the central laboratory within 24 h. Serum creatinine levels were measured using a Hitachi Automatic Analyzer 7600–210 (Hitachi, Tokyo, Japan).

### Renal function measurements

An estimated GFR (eGFR) was calculated using the Chronic Kidney Disease Epidemiology Collaboration equation  [27] The participants were grouped based on their eGFR levels as follows: stage 1 = eGFR ≥ 90 mL/min/1.73 m^2^, stage 2 = eGFR 60–89 mL/min/1.73 m^2^, stage 3a = eGFR 45–59 mL/min/1.73 m^2^, stage 3b = eGFR 30–44 mL/min/1.73 m^2^, and stage 4/5 = eGFR < 30 mL/min/1.73 m^2^[28] . Additionally, they were re-grouped according to their renal function stages (i.e., stages 1–2 and stages 3a–5).

### Hypertension, diabetes, and osteoporosis

Blood pressure measurements were performed on the right arm of the participants seated for at least 5 min, using a standard mercury sphygmomanometer. Three measurements were conducted for all participants at 5-min intervals, and the mean of the second and third measurements was used in the analysis. Hypertension was defined as systolic blood pressure ≥ 140 mmHg, diastolic blood pressure ≥ 90 mmHg, or use of antihypertensive medications independently of the blood pressure [29].

After a fasting period of at least 8 h, blood glucose was measured using a Hitachi Automatic Analyzer 7600–210 (Hitachi, Tokyo, Japan). Diabetes was defined as fasting glucose ≥ 126 mg/dL [30]. The participants with a known diagnosis of diabetes treated with antiglycemic agents and/or insulin, regardless of the fasting glucose level, were also included in the diabetes group.

Participants in this study underwent a comprehensive health interview conducted by a qualified medical doctor. The stratification into binary subgroups, distinguishing between non-osteoporosis and osteoporosis, was determined through a multifaceted evaluation process. This evaluation considered various factors, such as the participants' medical history, including any medications with potential effects on osteoporosis progression, and clinical assessments performed by the medical doctor. The inclusion of these factors in this assessment process might minimize the possibility of participants self-diagnosing their bone condition.

### Other variables

The subjects answered a self-reported questionnaire on age, socioeconomic variables (i.e., household income and educational level), and lifestyle variables (i.e., smoking status, alcohol consumption, and physical activity). Household income was categorized into quartile ranges based on the monthly average family equivalent income: low, lower middle, higher middle, and high. Educational level was divided into four groups: elementary school graduation or lower, middle school graduation, high school graduation, and college graduation or higher. Smoking status was divided into three groups. Non-smokers were defined as those who had never smoked in their lifetime, and past smokers were defined as those who had smoked in the past but did not smoke at the time of survey. Current smokers were defined as those who smoked daily or often at the time of survey. Heavy alcohol consumption was noted for women and men who had at least five and seven drinks, respectively, more than twice per week. Physical activity during work, transport, and leisure time was considered to assess the intensity and total time spent on physical activity per week. To meet the recommendations on physical activity for health, the participants were supposed to engage in at least 150 min/week of moderate-intensity physical activity, or 75 min/week of vigorous-intensity physical activity, or an equivalent combination of moderate- and vigorous-intensity physical activity while achieving ≥ 600 MET-minutes/week.

### Statistical analyses

All statistical analyses were conducted with SPSS 26.0, statistical software (SPSS Inc., Chicago, IL, USA) using the KNHANES sampling weights to calculate the representative estimates of the general Korean population. Data were analyzed using a complex survey design that considered stratified variables, cluster variables, and weighted variables. Statistical significance was set at *p* < 0.05. The participants included in the analysis were divided based on their muscle strength. The chi-square test was used to present categorical variables as frequency and percentage (%), and an independent *t *test was utilized to report continuous variables as mean and standard error. Logistic regression analysis for complex sampling adjusted for selected variables was utilized to assess the effects of osteoporosis (with the reference group including participants not diagnosed with osteoporosis) on muscle strength according to age (< 65 years and ≥ 65 years), BMI (< 18.5 kg/m^2^, ≥ 18.5 kg/m^2^ and < 25.0 kg/m^2^, and ≥ 25.0 kg/m^2^) and renal function (CKD stages 1–2 and CKD stages 3a-5) and the results were presented as odds ratios (ORs) with 95% confidence intervals (CIs).

## Results

Among the 26,855 subjects included in this study, LMS and NMS were reported in 4,135 (15.4%) and 22,720 (84.6%) subjects, respectively, and their characteristics are presented in Table 1. The mean ages of the subjects with LMS and NMS were 58.8 (0.5) and 45.0 (0.2) years, respectively. The mean dominant HGS levels of the subjects with LMS and NMS were 18.3 (0.1) and 32.0 (0.1) kg, respectively. Compared to subjects with NMS, the rates of hypertension (42.7% vs. 24.6%), diabetes (13.3% vs. 6.8%), osteoporosis (15.6% vs. 4.2%), and eGFR < 60 mL/min/1.73 m^2^ (8.3% vs. 1.7%) were higher in those with LMS. All the differences, except for the serum creatinine level, were statistically significant.

**Table 1:** Characteristics of the study population

Characteristic	LMS (N = 4,135)		NMS (N = 22,720)		*p* value
	% (SE)	Unweighted N	% (SE)	Unweighted N	
Age (years), mean (SE)	58.8 (0.5)	–	45.0 (0.2)	–	< 0.001
< 65	51.3 (1.1)	1,603	88.6 (0.3)	18,535	< 0.001
≥ 65	48.7 (1.1)	2,532	11.4 (0.3)	4,185	
Gender					
Men	42.8 (0.9)	1,724	51.1 (0.3)	10,181	< 0.001
Women	57.2 (0.9)	2,411	48.9 (0.3)	12,539	
Household income					
Low	30.8 (1.0)	1,249	23.6 (0.5)	5,218	< 0.001
Lower middle	25.0 (0.9)	1,043	25.1 (0.5)	5,676	
Higher middle	22.1 (0.8)	901	25.7 (0.4)	5,851	
High	22.2 (0.9)	924	25.5 (0.6)	5,904	
Educational level					
≤ Elementary school	39.1 (1.2)	1,947	10.3 (0.3)	3,328	< 0.00
Middle school	11.2 (0.6)	477	7.9 (0.2)	2,177	
High school	26.8 (0.9)	896	37.1 (0.5)	7,901	
≥ College	22.9 (1.0)	764	44.6 (0.6)	9,247	
Weight (kg), mean (SE)	59.7 (0.2)	–	66.2 (0.1)	–	< 0.001
Height (cm), mean (SE)	159.1 (0.2)	–	165.7 (0.1)	–	< 0.001
BMI (kg/m^2^), mean (SE)	23.5 (0.1)	–	24.0 (0.0)	–	< 0.001
< 18.5	6.7 (0.5)	237	3.8 (0.2)	780	< 0.001
≥ 18.5 and < 25.0	62.8 (0.9)	2,599	61.1 (0.4)	13,915	
≥ 25.0	30.5 (0.9)	1,265	35.1 (0.4)	7,998	
Smoking					
Never	64.3 (1.0)	2,643	55.8 (0.4)	13,436	< 0.001
Past	21.4 (0.7)	952	22.1 (0.3)	4,958	
Current	14.3 (0.7)	502	22.1 (0.4)	4,248	
Heavy alcohol drinking	6.4 (0.5)	229	13.8 (0.3)	2,788	< 0.001
Hypertension	42.7 (1.0)	2,026	24.6 (0.4)	6,557	< 0.001
Diabetes	13.3 (0.6)	578	6.8 (0.2)	1,760	< 0.001
Osteoporosis	15.6 (0.7)	766	4.2 (0.1)	1,420	< 0.001
Physical activity					
< 600 MET-minutes/wk	65.8 (1.0)	2,803	50.8 (0.5)	12,121	< 0.001
≥ 600 MET-minutes/wk	34.2 (1.0)	1,257	49.2 (0.5)	10,526	
Right HGS (kg), mean (SE)	18.3 (0.1)	–	31.9 (0.1)	–	< 0.001
Left HGS (kg), mean (SE)	18.7 (0.1)	–	30.4 (0.1)	–	< 0.001
Dominant HGS (kg), mean (SE)	18.3 (0.1)	–	32.0 (0.1)	–	< 0.001
Creatinine (mg/dL), mean (SE)	0.8 (0.0)	–	0.8 (0.0)	–	0.738
eGFR (mL/min/1.73 m^2^), mean (SE)	90.4 (0.5)	–	99.1 (0.2)	–	< 0.001
CKD Stages 1–2	91.7 (0.5)	3,489	98.3 (0.1)	21,631	< 0.001
CKD Stages 3a–5	8.3 (0.5)	401	1.7 (0.1)	550	

LMS, low muscle strength; NMS, normal muscle strength; SE, standard error; BMI, body mass index; MET, metabolic equivalents; HGS, hand grip strength; eGFR, estimated glomerular filtration rate; CKD, chronic kidney disease

In men, LMS defined as HGS < 28.9 kg, and NMS as HGS ≥ 28.9 kg; in women, LMS defined as HGS < 16.8 kg, and NMS as HGS ≥ 16.8 kg

CKD Stages 1–2 defined as eGFR ≥ 60 mL/min/1.73 m^2^, and CKD Stages 3a-5 as eGFR < 60 mL/min/1.73 m^2^

The association between osteoporosis and the prevalence of LMS in all subjects was assessed using logistic regression analysis, and the results are summarized in Table 2. The osteoporotic subjects had a higher prevalence rate for LMS than those without osteoporosis after adjusting for age (OR, 1.684; 95% CI, 1.500–1.890). After further adjusting for other factors, including age, significant differences were still observed. Similar tendencies were also observed in the subjects aged < 65 years and ≥ 65 years when analyzed by age; however, the prevalence rate of LMS was higher in the former than in the latter group.

**Table 2:** Odds ratios for muscle strength in total subjects

Subgroups	LMS		NMS		Model 1 [OR (95% CI)]	Model 2 [OR (95% CI)]	Model 3 [OR (95% CI)]	Model 4 [OR (95% CI)]	Model 5 [OR (95% CI)]
	% (SE)	Unweighted N	% (SE)	Unweighted N					
Total									
Non-osteoporosis	84.4 (0.7)	3,369	95.8 (0.1)	21,300	1	1	1	1	1
Osteoporosis	15.6 (0.7)	766	4.2 (0.1)	1,420	4.236 (3.788–4.738)	1.684 (1.500–1.890)	1.559 (1.380–1.761)	1.539 (1.359–1.743)	1.338 (1.176–1.524)
< 65 years									
Non-osteoporosis	95.5 (0.6)	1,513	97.9 (0.1)	18,005	1	1	1	1	1
Osteoporosis	4.5 (0.6)	90	2.1 (0.1)	530	2.148 (1.637–2.818)	1.921 1.444–2.555)	1.685 (1.259–2.255)	1.647 (1.227–2.211)	1.399 (1.038–1.885)
≥ 65 years									
Non-osteoporosis	72.6 (1.0)	1,856	79.8 (0.7)	3,295	1	1	1	1	1
Osteoporosis	27.4 (1.0)	676	20.2 (0.7)	890	1.486 (1.301–1.696)	1.334 (1.153–1.544)	1.458 (1.244–1.708)	1.404 (1.195–1.648)	1.314 (1.105–1.563)

LMS, low muscle strength; NMS, normal muscle strength; SE, standard error

Odds ratios with adjustments using logistic regression models

Model 1: unadjusted; Model 2: adjusted for age; Model 3: adjusted for age and sex; Model 4: adjusted for age, sex, and BMI; Model 5: adjusted for age, sex, household income, educational level, BMI, smoking, heavy alcohol drinking, hypertension, diabetes, physical activity, and eGFR

The results of the logistic regression analysis for all the subjects according to BMI are presented in Table 3. Subjects with osteoporosis and BMI < 18.5 kg/m^2^ had a higher prevalence rate of LMS after adjusting for age compared to those without osteoporosis and with BMI < 18.5 kg/m^2^ (OR, 1.872; 95% CI, 1.043–3.359). Similar tendencies were exhibited after adjusting for age in subjects of the other two groups; however, the ORs of LMS in these groups were reduced. In the subjects aged < 65 years, the ORs of LMS were higher in the osteoporotic subjects than in those without osteoporosis when adjusted for age and sex; however, the OR was not statistically significant in the subjects with BMI ≥ 25 kg/m^2^. In subjects aged ≥ 65 years, the ORs of LMS were also higher in the osteoporotic subjects than in those without osteoporosis when adjusted for age and sex, but the OR was not statistically significant in the subjects with BMI < 18.5 kg/m^2^.

**Table 3:** Odds ratios for muscle strength in total subjects according to age and BMI

Subgroups	LMS		NMS		Model 1 [OR (95% CI)]	Model 2 [OR (95% CI)]	Model 3 [OR (95% CI)]	Model 4 [OR (95% CI)]
	% (SE)	Unweighted N	% (SE)	Unweighted N				
Total								
BMI < 18.5 kg/m^2^								
Non-osteoporosis	86.7 (2.4)	197	97.4 (0.5)	745	1	1	1	1
Osteoporosis	13.3 (2.4)	40	2.6 (0.5)	35	5.697 (3.217–10.090)	1.872 (1.043–3.359)	2.532 (1.351–4.743)	2.414 (1.242–4.689)
18.5 kg/m^2^ ≤ BMI < 25 kg/m^2^								
Non-osteoporosis	84.5 (0.8)	2,130	95.6 (0.2)	13,007	1	1	1	1
Osteoporosis	15.5 (0.8)	469	4.4 (0.2)	908	3.962 (3.434–4.572)	1.549 (1.330–1.803)	1.497 (1.275–1.758)	1.309 (1.099–1.558)
BMI ≥ 25 kg/m^2^								
Non-osteoporosis	84.2 (1.2)	1,021	96.1 (0.2)	7,524	1	1	1	1
Osteoporosis	15.8 (1.2)	244	3.9 (0.2)	474	4.613 (3.793–5.610)	1.771 (1.444–2.172)	1.518 (1.225–1.880)	1.311 (1.045–1.646)
< 65 years								
BMI < 18.5 kg/m^2^								
Non-osteoporosis	94.3 (1.7)	111	98.4 (0.5)	703	1	1	1	1
Osteoporosis	5.7 (1.7)	9	1.6 (0.5)	17	3.687 (1.540–8.825)	3.481 (1.287–9.418)	4.353 (1.581–11.985)	4.076 (1.610–10.319)
18.5 kg/m^2^ ≤ BMI < 25 kg/m^2^								
Non-osteoporosis	95.4 (0.8)	970	97.6 (0.1)	11,113	1	1	1	1
Osteoporosis	4.6 (0.8)	54	2.4 (0.1)	363	1.987 (1.384–2.851)	1.660 (1.133–2.434)	1.524 (1.033–2.247)	1.356 (0.914–2.011)
BMI ≥ 25 kg/m^2^								
Non-osteoporosis	96.3 (0.8)	430	98.3 (0.2)	6,171	1	1	1	1
Osteoporosis	3.7 (0.8)	26	1.7 (0.2)	149	2.160 (1.316–3.546)	1.940 (1.156–3.254)	1.528 (0.899–2.599)	1.130 (0.636–2.007)
≥ 65 years								
BMI < 18.5 kg/m^2^								
Non-osteoporosis	72.8 (4.5)	86	73.6 (5.0)	42	1	1	1	1
Osteoporosis	27.2 (4.5)	31	26.4 (5.0)	18	1.042 (0.512–2.119)	1.052 (0.494–2.240)	1.304 (0.540–3.153)	1.024 (0.351–2.986)
18.5 kg/m^2^ ≤ BMI < 25 kg/m^2^								
Non-osteoporosis	72.7 (1.3)	1,160	78.8 (0.9)	1,894	1	1	1	1
Osteoporosis	27.3 (1.3)	415	21.2 (0.9)	545	1.401 (1.175–1.669)	1.312 (1.088–1.582)	1.476 (1.198–1.820)	1.404 (1.116–1.767)
BMI ≥ 25 kg/m^2^								
Non-osteoporosis	72.9 (1.9)	591	81.4 (1.1)	1,353	1	1	1	1
Osteoporosis	27.1 (1.9)	218	18.6 (1.1)	325	1.624 (1.289–2.046)	1.371 (1.072–1.754)	1.350 (1.035–1.759)	1.208 (0.922–1.583)

LMS, low muscle strength; NMS, normal muscle strength; SE, standard error

Odds ratios with adjustments using logistic regression models

Model 1: unadjusted; Model 2: adjusted for age; Model 3: adjusted for age and sex; Model 4: adjusted for age, sex, household income, educational level, smoking, heavy alcohol drinking, hypertension, diabetes, physical activity, and eGFR

The results of the logistic regression analysis for all subjects according to renal function are summarized in Table 4. Compared to the non-osteoporotic subjects with eGFR ≥ 60 mL/min/1.73 m^2^, those with osteoporosis and eGFR ≥ 60 mL/min/1.73 m^2^ had a higher prevalence rate for LMS after controlling for age and sex (OR, 1.630; 95% CI, 1.427–1.862). This tendency was still present after further adjusting for other factors in subjects with eGFR ≥ 60 mL/min/1.73 m^2^. However, the ORs of LMS for subjects with eGFR < 60 mL/min/1.73 m^2^ were not statistically significant after controlling for confounders. In subjects aged < 65 years, the ORs of LMS were higher in subjects with osteoporosis than in those without osteoporosis when adjusted for age and sex, regardless of the renal function status. However, the OR of the subjects with eGFR < 60 mL/min/1.73 m^2^ was higher than that of their counterparts. No statistically significant difference was observed between subjects aged ≥ 65 years and those with eGFR < 60 mL/min/1.73 m^2^, whereas the OR of LMS in subjects aged ≥ 65 years and those with eGFR ≥ 60 mL/min/1.73 m^2^ was higher in subjects with osteoporosis than in those without osteoporosis when controlling for age and sex.

**Table 4:** Odds ratios for muscle strength in total subjects according to age and renal function

Subgroups	LMS		NMS		Model 1 [OR (95% CI)]	Model 2 [OR (95% CI)]	Model 3 [OR (95% CI)]	Model 4 [OR (95% CI)]	Model 5 [OR (95% CI)]
	% (SE)	Unweighted N	% (SE)	Unweighted N					
Total									
CKD Stages 1–2									
Non-osteoporosis	85.4 (0.7)	2,863	96.0 (0.1)	20,349	1	1	1	1	1
Osteoporosis	14.6 (0.7)	626	4.0 (0.1)	1,282	4.157 (3.674–4.703)	1.770 (1.560–2.007)	1.630 (1.427–1.862)	1.608 (1.405–1.842)	1.402 (1.224–1.606)
CKD Stages 3a-5									
Non-osteoporosis	79.1 (2.3)	323	85.0 (1.6)	461	1	1	1	1	1
Osteoporosis	20.9 (2.3)	78	15.0 (1.6)	89	1.495 (1.026–2.179)	1.055 (0.707–1.574)	1.024 (0.658–1.596)	0.984 (0.629–1.539)	0.943 (0.596–1.491)
< 65 years									
CKD Stages 1–2									
Non-osteoporosis	95.6 (0.6)	1,449	97.9 (0.1)	17,512	1	1	1	1	1
Osteoporosis	4.4 (0.6)	85	2.1 (0.1)	513	2.127 (1.606–2.818)	1.910 (1.424–2.561)	1.674 (1.240–2.259)	1.634 (1.206–2.212)	1.350 (1.000–1.824)
CKD Stages 3a-5									
Non-osteoporosis	86.0 (2.6)	17	96.4 (1.7)	124	1	1	1	1	1
Osteoporosis	14.0 (2.6)	2	3.6 (1.7)	5	4.434 (1.570–12.522)	4.357 (1.540–12.326)	3.572 (1.213–10.519)	3.651 (1.220–10.924)	2.340 (0.694–7.891)
≥ 65 years									
CKD Stages 1–2									
Non-osteoporosis	71.9 (1.2)	1,414	79.7 (0.8)	2,837	1	1	1	1	1
Osteoporosis	28.1 (1.2)	541	20.3 (0.8)	769	1.532 (1.322–1.776)	1.357 (1.153–1.596)	1.496 (1.254–1.786)	1.446 (1.210–1.729)	1.405 (1.164–1.697)
CKD Stages 3a-5									
Non-osteoporosis	78.6 (2.4)	306	79.9 (2.1)	337	1	1	1	1	1
Osteoporosis	21.4 (2.4)	76	20.1 (2.1)	84	1.085 (0.743–1.586)	0.977 (0.660–1.445)	0.991 (0.635–1.547)	0.934 (0.597–1.461)	0.876 (0.562–1.365)

LMS, low muscle strength; NMS, normal muscle strength; SE, standard error

Odds ratios with adjustments using logistic regression models

Model 1: unadjusted; Model 2: adjusted for age; Model 3: adjusted for age and sex; Model 4: adjusted for age, sex and BMI; Model 5: adjusted for age, sex, household income, educational level, BMI, smoking, heavy alcohol drinking, hypertension, diabetes, and physical activity

## Discussion

In this study, using representative and reliable data, we evaluated the associations between osteoporosis and HGS, a surrogate marker of muscular strength, among Korean adults. The major results of this study indicated that LMS tends to be more prevalent in osteoporotic subjects than in healthy individuals. In particular, the prevalence of LMS was likely to be higher in underweight subjects than in subjects with a normal weight, overweight, or obese subjects. To the best of our knowledge, studies on the relationship between osteoporosis and HGS have rarely been conducted on Korean adults with consideration of additional contributing factors (i.e., BMI, age, and renal function) for osteoporosis. Therefore, the findings of this study could be used to guide the development of healthcare strategies for the management of patients with poor bone health.

The overall prevalence of LMS was 1.684 times higher in osteoporotic subjects than in their healthy counterparts after adjusting for age, and the pattern was still observed after further adjustment for other variables, although the association between osteoporosis and HGS was attenuated. This is consistent with findings of some previous studies [31–35]. Luo et al. reported that in the general US population, HGS was positively associated with BMD levels of femoral neck and lumbar spine regardless of sex and menopausal status [31]. According to the cross-sectional study conducted by Lin et al. on Chinese post-menopausal women and men aged ≥ 50 years, HGS was positively linked with BMD in both sexes, and it was also negatively associated with osteoporosis in men (OR, 0.88; 95% CI, 0.83–0.94) as well as in women (OR, 0.96; 95% CI, 0.92–0.98) [32]. McGrath et al. also reported that the prevalence odds of osteoporosis in the general US population aged ≥ 40 years were reduced by approximately 6% for men and 10% for women as HGS increased by every 0.1 kg [34]. Taken together, higher levels of HGS are likely to increase BMD levels and ultimately decrease the prevalence of osteoporosis. These results may be explained by the effect of myokines (e.g., irisin) secreted from the muscles on bone health [17]. Irisin level is positively related to BMD status, and its low level may result in an increased prevalence of hip fracture [36]. Similar patterns were also observed in the subgroup analyses stratified by age. However, the association between LMS and osteoporosis was weaker in subjects aged ≥ 65 years than in their counterparts, which may be explained by age-associated decreases in muscle and bone due to decreased irisin levels [36].

Underweight subjects with osteoporosis had a higher prevalence rate of LMS than subjects with a normal weight, as well as overweight and obese subjects with osteoporosis after adjusting for various variables including age. The result could be, to some extent, explained by the synergistic effect of BMI and osteoporosis on HGS. Interim gain or a small change in BMD was observed in men and women who gained > 5% of their baseline weight compared to those with ≤ 5% change from their baseline weight during the observation period [18, 19]. A decrease in BMD secondary to weight loss was shown in peri- and post-menopausal women and older men, and the annual rate of their bone loss was > 2 times faster than that of bone loss in individuals with a stable weight [18]. Higher BMI is likely to have a protective effect on BMD loss. HGS was also positively associated with BMI in both men and women; in particular, this association was more prominent in the obese group than in the overweight group [37]. Taken together, BMI can positively affect HGS directly or indirectly by increasing BMD. However, in the subgroup analyses stratified by age, this tendency was observed only in subjects aged < 65 years. This may suggest that the effect of aging on HGS is relatively stronger than that of BMI, although other contributing factors of low HGS should be considered. In a previous study [38], the peak of HGS was reached at an age of approximately 40 years, and HGS remained stable or was slightly reduced in the period between 40 and 50 years, following a rapid reduction after the age of 50.

The prevalence of LMS was significantly higher in osteoporotic subjects with eGFR ≥ 60 mL/min/1.73 m^2^ than in non-osteoporotic subjects with eGFR ≥ 60 mL/min/1.73 m^2^. This tendency remained even after adjusting for various variables. However, this tendency disappeared in subjects with eGFR < 60 mL/min/1.73 m^2^ when adjusted for the variables. This result could be partially explained by the hypothesis that the renal function has a relatively stronger effect on HGS than on the osteoporosis status. Kidney insufficiency can contribute to various clinical problems such as anemia, decrease in hemoglobin level, presence of proteinuria, protein hypercatabolism, and disorders of mineral and bone metabolism, thereby ultimately leading to poor muscle strength and mass [39]. Hiraki and colleagues reported that significantly lower HGS was observed in patients with CKD stage 4 or 5 compared with those with CKD stage 2 or 3 [40]. The reduction in average HGS was shown from 35.2 kg among patients with CKD stage 2 to 22.4 kg among those with CKD stage 5 [40]. Even mildly reduced kidney function was also associated with sarcopenia, defined as a progressive loss of muscle strength and mass [41]. Interestingly, the prevalence of LMS was significantly higher in osteoporotic subjects aged < 65 years and with eGFR < 60 mL/min/1.73 m^2^ compared to non-osteoporotic subjects aged < 65 years and with eGFR < 60 mL/min/1.73 m^2^. However, a similar result was not observed among subjects aged ≥ 65 years and with eGFR < 60 mL/min/1.73 m^2^. This may indicate that aging is more strongly associated with HGS than with reduced kidney function.

In the present study, the relationship between osteoporosis and HGS was evaluated, and we found that osteoporosis had a significant effect on HGS. Additionally, aging, BMI, and renal function were found to be critical determinants of the HGS status, as also shown in previous studies. Musculoskeletal aging has many causes, such as age-related changes in body composition, inflammation, and hormonal imbalance, which are associated with osteoporosis and sarcopenia (loss of muscle mass and strength). In particular, the decline in physical activity may lead to osteoporosis and sarcopenia, which are typical features of aging, and these two diseases often induce a frailty syndrome. Muscle and bone tissues have been progressively identified as endocrine target organs and endocrine organs themselves, interacting via paracrine and endocrine signals and modulating their development and function [42–44]. During growth, BMD closely correlates with muscle mass, and growing evidence shows that osteoporosis and sarcopenia share many common pathophysiological factors, including age-related chronic inflammation, hormonal imbalance, anabolic or catabolic molecules released by the skeletal muscle or by the bone cells, changes in body composition, and physical impairment [45]. Interestingly, various tissue-specific factors secreted by the muscle tissue, including tumor necrosis factor-α, interleukin (IL)-6, IL-15, reactive oxygen species, irisin, and myostatin, are linked to the pathogenesis of sarcopenia and are also modulators of bone remodelling, and thus are related to osteoporosis [44, 46]. Moreover, previous studies have reported the mechanism between bones and muscles has bidirectional relationship. Muscles can affect bones and influences from bone to muscle also exist. Previous study showed that bone marrow mesenchymal stromal cells stimulate myoblast proliferation through vascular endothelial growth factor (VEGF) from mesenchymal stromal cells, suggesting that bone mesenchymal cells can influence muscle cells [47]. Osteocytes are abundant in bone tissues and noted as endocrine cells that affect various organs, such as parathyroid glands and kidney. A recent study revealed that mechanically loaded osteocytes produce various factors, such as insulin-like growth factor-1, mechano growth factor, VEGF and hepatocyte growth factor, which may be anabolic and metabolic factors regulating muscle mass [48]. Moreover, osteocytes produce factors such as Wnt3a and prostaglandin E2 that support myogenesis and muscle function [49]. Furthermore, obesity can also affect sarcopenia and osteoporosis through the interaction between the adipose tissue, bone, and muscle. Age-related augmented visceral fat and muscle fat infiltration boost insulin resistance and inflammation, which, through a vicious cycle, affects skeletal and muscle metabolism alterations and dysregulation leading to osteoporosis and sarcopenia [50]. Indeed, several studies have demonstrated that obesity is linked to sarcopenia, osteoporosis, and frailty due to adipose tissue involvement in the complex bone-muscle interaction [43, 51]. Meanwhile, reduction of muscle mass in CKD may result in a negative balance of protein homeostasis, which is involved in increased catabolism and reduced anabolism of the muscle tissue and impaired muscle regeneration. In addition, muscle regeneration and size can be affected by myostatin, a negative regulator of skeletal muscle mass, and myostatin is upregulated in the blood of patients with CKD [52]. In CKD patients, the renin–angiotensin–aldosterone system becomes elevated, which hinders muscle regeneration through the ubiquitin–proteasome proteolytic pathway that degrades ubiquitinated proteins through the activation of nuclear forkhead box members. [52, 53]

The present study has some limitations that should be considered when interpreting the results. First, it was difficult to clearly conclude causality between osteoporosis and the prevalence of LMS due to the cross-sectional design of this study. Second, almost all the variables measured at a single time point were used to determine the effects of osteoporosis on the prevalence of LMS, which is likely to have a negative effect on data accuracy. Third, the sociodemographic characteristics of the study population were obtained through a survey, which might have led to recall bias, at least in some cases. Fourth, the overall prevalence of LMS was likely to be underestimated to some extent, since the subjects without three HGS measurements in both hands were excluded. However, this process was unlikely to significantly affect the study results because missing measurements were recorded randomly. Finally, the osteoporotic status of the participants was not assessed using objective measurements (e.g., BMD) accurately representing their bone health, since the data related to the diagnosis of osteoporosis by a doctor were obtained through a health interview. This might have influenced the assessment of the prevalence of osteoporosis.

## Conclusion

Our findings indicate that osteoporosis is likely to contribute to an increased prevalence of LMS in terms of HGS. Aging, BMI, and renal function also had significant effects on the association between osteoporosis and LMS. This association is likely to facilitate the development of better strategies of bone health estimation in the clinic.

## Data Availability

The datasets used for this study are available from the corresponding author on reasonable request.
